# Effect of polyhydroxy-terminated PAMAM dendrimer on dentin matrix metalloproteinases within the hybrid layers

**DOI:** 10.1186/s12903-023-02841-2

**Published:** 2023-03-11

**Authors:** Yu Luo, Ruirui Si, Yuan He, Mengmeng Wang, Yingying Yu, Xin Huang, Rong Huang, Yingyi Huang, Yang Luo, Wei Jin, Yaping Gou

**Affiliations:** 1 School/Hospital of Stomatology, Lanzhou University, Lanzhou, Gansu Province PR China; 2 Key Laboratory of Dental Maxillofacial Reconstruction and Biological Intelligence Manufacturing, Lanzhou, Gansu Province PR China

**Keywords:** PAMAM-OH, Collagen fibril, Dentin, Matrix metalloproteinases, Resin-dentin bonds

## Abstract

**Background:**

Intrafibrillar remineralization within the hybrid layers (HLs) has recently attracted extensive attention in achieving durable resin-dentin bonds. The polyhydroxy-terminated poly(amidoamine) dendrimer (PAMAM-OH) at fourth generation becomes a desirable candidate to induce intrafibrillar remineralization to protect exposed collagen fibrils within HLs based on the size exclusion effect of fibrillar collagen. However, the remineralization process in vivo is time-consuming, during which the exposed collagen fibrils are vulnerable to enzymatic degradation, resulting in unsatisfactory remineralization. Thereby, if PAMAM-OH itself possesses concomitant anti-proteolytic activity during the induction of remineralization, it would be very beneficial to obtain satisfactory remineralization.

**Methods:**

Binding capacity tests using adsorption isotherm and confocal laser scanning microscopy (CLSM) were performed to assess if the PAMAM-OH had adsorption capacity on dentin. Anti-proteolytic testings were detected by MMPs assay kit, in-situ zymography and ICTP assay. Adhesive infiltration of resin-dentin interface and tensile bond strength before and after thermomechanical cycling were implemented to assess if the PAMAM-OH adversely affected resin-dentin bonds.

**Results:**

Anti-proteolytic testings performed using MMPs assay kit, in-situ zymography and ICTP assay indicated that PAMAM-OH inhibited exogenous soluble MMP-9 as well as had inhibitory effect on the endogenous proteases. Adhesive infiltration of resin-dentin interface and tensile bond strength before and after thermomechanical cycling were implemented to indicate that the PAMAM-OH pretreatment had no adverse effects on immediate dentin bonding and prolonged the durability of resin-dentin bonds.

**Conclusions:**

PAMAM-OH possesses anti-proteolytic activity and prevents exposed collagen fibrils within HLs from degradation, which lays the foundation for the satisfactory intrafibrillar remineralization induced by PAMAM-OH within HLs to achieve durable resin-dentin bonds in the next work.

## Introduction

In recent years, composite resin becomes the most popular treatment for repairing dental defects due to its excellent aesthetics. Resin-dentin bonds mainly depend upon sufficient infiltration of adhesive into collagen matrix to produce the hybrid layers (HLs) [1, 2]. Actually, adhesive fails to completely infiltrate into the collagen matrices [3], causing a series of issues. The most striking thing is that the exposed collagen fibrils are degraded by endogenous matrix metalloproteinases (MMPs) that can be activated by the acid-etching phase of dentin bonding [4–7]. Additionally, these unprotected collagen fibrils at the bottom of the HLs are prone to fatigue breakdown after repetitive loads due to the loss of intrafibrillar minerals [8]. These adverse factors deteriorate the durability of resin-dentin bonding [9–11].

Currently, several strategies have been used to address the aforementioned issues. For example, chlorhexidine, as the most commonly used MMPs inhibitors, has been widely used to inhibit collagenolytic activity within the HLs [6]. However, chlorhexidine is water-soluble and only binds to demineralized dentin through weak electrostatic interaction, and its long-term anti-proteolytic activity is compromised [12]. Another approach is to use collagen cross-linking agents such as riboflavin. The use of cross-linkers has been confirmed to improve the collagen stiffness, tensile strength and compressive modulus by strengthening the interfacial layer through additional hydrogen bonds and formation of covalent intra- and intermolecular crosslinks, and by inhibiting dentin MMPs via masking the cleavage sites of collagen [13–21]**.** Although cross-linker can maintain the integrity of the mineral-denuded collagen matrices, the water-filled, resin-sparse and flaccid collagen fibrils are prone to cyclic fatigue breakdown after repetitive loads [8].

The remineralization of demineralized dentin collagen within HLs has been proposed as a strategy superior to the aforementioned methods. By inducing the deposition of mineral crystals in intrafibrillar compartments of collagen fibril, the biomimetic remineralization makes the water in intrafibrillar compartments be replaced by apatite and fossilizes dentin proteases [22–24], which restores the flaccid collagen fibril to the natural mineralization state and improves the resistance of collagen matrices to cyclic fatigue rupture and enzymatic corruption. The process of biomimetic remineralization mainly depends on three factors, including non-collagenous proteins (NCPs), collagen scaffold and extraneous calcium and phosphorus. NCPs are very important for the modulation of the biomineralization process to biomimetically induce the hierarchical intrafibrillar mineralization [25]. However, it is not easy to obtain native NCPs and enable them to be commercially available. Therefore, many NCPs analogues with well-defined steric structure have been studied to mimic the functions of natural NCPs [26].

Poly(amidoamine) (PAMAM) dendrimers are kind of hyperbranched polymeric macromolecules consisting of a core, internal cavity, highly branched structure and functional groups on the exterior [27]. With a great number of reactive groups on their exterior, well-defined size and symmetrical structure, PAMAM dendrimers enable themselves to be a desirable candidate to mimic the natural NCPs [28]. The application of PAMAM dendrimers in the field of biomineralization has become hot research recently, and PAMAM dendrimers bearing different types of terminal groups on the surface are able to modulate the size, morphology and dimension of mineral crystals in vitro [25, 29, 30]. Polyhydroxy-terminated PAMAM (PAMAM-OH) dendrimer has been applied to act as NCPs to induce biomimetic remineralization on the dentinal tubule due to its relatively cheap cost and superior biocompatibility [30]. However, there has been no report whether PAMAM-OH can induce intrafibrillar mineralization of dentin.

Furthermore, dentin collagen matrix is the fundamental scaffold for the growth of dentin minerals, which plays a significant role in biomimetic remineralization [2]. However, the remineralization process in vivo is time-consuming, during which the denuded collagen fibrils are vulnerable to enzymatic degradation, resulting in unsatisfactory remineralization. Therefore, how to protect the exposed collagen fibrils within HLs from degrading of MMPs during remineralization becomes the urgent problem for the clinical application of biomimetic remineralization [31, 32]. Obviously, if NCPs analogues (e. g. PAMAM-OH) themselves possess concomitant anti-proteolytic activity during the induction of remineralization, it would be very beneficial to obtain satisfactory remineralization.

Thus, the present study aimed to explore whether PAMAM-OH can inhibit soluble and dentin-bound MMPs using functional enzyme activity assays and in-situ zymography. Besides, the effects of PAMAM-OH on resin-dentin interface before and after thermal cycling were also evaluated. The null hypotheses tested were as follows: (a) PAMAM-OH does not inhibit the activity of endogenous MMPs; (b) PAMAM-OH has adverse impact on the resin-dentin bonds.

## Methods

### Cytotoxicity

The cytotoxicity of PAMAM-OH on the viability of human dental pulp stem cells (HDPCs) and mouse fibroblast cells L929 was assessed using Cell Counting Kit-8 (CCK-8). HDPCs and L929 were cultured as described below. Following the patients’ informed consent, three teeth were collected and transported to the lab. Pulp tissues were carefully extracted by blunt non-cutting forceps and immersed into collagenase/dispase solution for 1.5 h. The cells were subsequently resuspended in Dulbecco’s Modified Eagle’s Medium (DMEM) containing necessary supplements. The seeded cells were grown at 37 °C in a humidified incubator with 5% CO_2_ until achieving 85% confluency.

The third passage cells were seeded in a 96-well plate at a density of 10^4^ cells per well and incubated for 24 h. Then the medium was replaced with fresh medium containing different concentrations of PAMAM-OH. After 24 h of incubation, the required CCK-8 solution was added to each well according to the instructions. The micro titration plate was transferred to a constant temperature incubator for 4 h under light-proof conditions. The absorbance was measured at 570 nm.

### Characterization of adsorption capacity of PAMAM-OH on demineralized dentin

#### Adsorption capacity of PAMAM-OH on hydroxyapatite (HA) powders

The PAMAM-OH solutions with concentrations from 1 to 10 mg/ml (1, 2, 4, 6, 8, 10 mg/ml) were firstly prepared. HA powders (100 mg) were immersed in 1 ml of each PAMAM-OH solution, stirred in 10 ml tubes at 37 °C overnight, and subsequently centrifuged at 10000 rpm for 3 min. The supernatant was collected, filtered and determined at a wavelength of 282 nm to assess the amount of PAMAM-OH. The amount of PAMAM-OH adsorbed on HA powders was calculated by the decrease of PAMAM-OH in the supernatant.

#### Synthesis of FITC-labeled PAMAM-OH

To visually observe whether PAMAM-OH can bind to demineralized dentin, a threefold molar amount of FITC solution (in acetone) and G4-PAMAM-OH aqueous solution was mixed and stirred in the dark for 24 h. Then the mixture was air-sparged to remove excess acetone, dialyzed against deionized water overnight and filtered to separate free FITC. The conjugate was finally lyophilized for subsequent experiment.

#### Assessment adsorption capacity of PAMAM-OH on demineralized dentin

FITC-labeled PAMAM-OH solution (8 mg/mL, 50 μL) and free FITC solution were separately applied to the demineralized dentin disk. After being dried at room temperature, each sample was washed with deionized water and dried again. The specimens were scanned by CLSM (LSM 780, Carl Zeiss). An imaging software (Image-Pro Plus 6.0, MD, USA) was used to quantify the green fluorescence intensity that represents the adsorption capacity of PAMAM-OH to demineralized dentin.

### Inhibition of gelatinolytic activity

#### Soluble rhMMP-9

Various concentrations of PAMAM-OH solutions as test agents (0.25, 0.5, 1, 2.5, 5, and 10 mg/mL) were used to test. The purified recombinant human (rh) MMP-9 and the Sensolyte Generic MMP assay kit (all from AnaSpec Inc.) were used to examine the effect of PAMAM-OH on soluble rhMMP-9. The thiopeptolide (substrate) in the MMP assay kit was decomposed by MMPs to gradually release a colored product, which can be quantified at a wavelength of 412 nm in a 96-well plate.

The kit inhibitor (GM6001) in the MMP kit was tested as the ‘inhibitor control’, and the “positive control” included the other concentrations of PAMAM-OH. The inhibitory effect of PAMAM-OH on rhMMP-9 was expressed as percentages of the obtained ‘positive control’ concentration, which was regarded as the maximum concentration.

#### In-situ zymography of resin-dentin interfaces

Following the patients’ informed consent, forty teeth from each of the control and PAMAM-OH group were cut into segments and used for in-situ zymography. The dentin segments were acid-etched with 37% phosphoric acid gel, washed with deionized water and gently air-dried. Specimens were then randomly divided into 2 groups (*n* = 20). The control was pre-treated with deionized water for 60 s. The experimental sample was pre-treated with 8 mg/mL PAMAM-OH for 60 s. A 2-mm-thick layer of flowable resin composite was placed on the dentin bonded with dye-doped adhesive and light-cured. Then, specimens from each group were divided into 2 subgroups (*n* = 10). The samples were stored in deionized water for 48 h, labelled as T0. The other samples were thermo-mechanically challenged, corresponding to 1 y of intraoral use [33], labelled as T1. Each bonded specimen was cut into 1-mm-thick slices under a low-speed cutter and finally polished by wet silicon carbide to grind into approximately 50 μm-thick sections. All these sections contain the dentin-resin interface.

An important initiating factor for the degradation of collagen fibers is the activation of MMP, so we performed in-situ zymography using quenched fluorescein-conjugated gelatin (E-12055; Molecular Probes) to identify the active site of MMP in the hybrid layer [34]. The prepared fluorescent mixture as an MMP substrate was pipetted onto each glass slide according to the previous procedure and covered with a coverslip. The glass slides were incubated in a 100% humidity chamber and kept away from light. CLSM (LSM780; Carl Zeiss) was used to evaluate endogenous gelatinolytic activity. Image channels were set at 488/530 nm. Gelatinolytic activity was quantified using ImageJ and expressed as a percentage of green fluorescence in the HLs.

#### Measurement of released ICTP

Cross-linked carboxyterminal telopeptide (ICTP) is released after type I collagen is degraded by MMPs. Thus, the amount of ICTP reflects MMPs activity. Fifty teeth were collected and subjected to two parallel incisions perpendicularly to the longitudinal axis of the tooth to obtain dentin samples. The excess enamel, pulp soft tissues and cementum were removed. The dentin samples were placed in a stainless-steel spiral jacking tubes and frozen in liquid nitrogen for 4 min before being transferred to a mill for repeated grinding. This process was repeated to ensure the powder with a diameter of less than 40 μm. The dentin powder was immersed in 10 wt% phosphoric acid (pH = 1.0) at 4 °C for 8 h to guarantee complete demineralization. The demineralized dentin powder was washed with deionized water three times and lyophilized. These samples were then divided into two different groups (control group and PAMAM-OH group), and each group was divided into five 10 mg aliquots subpackaged in microcentrifuge tubes. Each 10 mg aliquot of powder was immersed in deionized water or artificial saliva respectively. After 2 weeks of incubation, 50 μl of the incubation medium was gathered to quantify solubilized collagen fragments. The measurement was performed with a spectrometer (Beckman Coulter, Inc., Indianapolis, IN, USA) at the absorbance of 450 nm and the amount of ICTP was calculated according to the standard curve constructed using the standards of known concentrations provided in the ICTP ELISA kit (Catalogue no. CSB-E11224 h, Cusabio, Wuhan, China).

### Analysis of the effect of PAMAM-OH on resin-dentin interface

#### Adhesive permeation through resin-dentin interface

Ten teeth from each of the control and PAMAM-OH groups were selected for permeability evaluation. 2.5 mm-thick midcoronal dentin surface was obtained with slow-speed saw. Fluorescent adhesive was prepared by mixing fluorescein dye with adhesive. The dentin segments to be bonded were adhered to perforated Plexiglass block. The assembly was connected to a polyethylene tubing via an 18-gauge stainless steel tube. The polyethylene tubing was immersed into a column of 0.1% blue fluorescent dye solution (Alexa Fluor™ 405, λ_excitation_/λ_emission_ = 401/421 nm) oriented 20 cm above the Plexiglass block to simulate the pulpal pressure. Water pressure was delivered to the bonded interface through the dentinal tubules during the acid-etching process, pretreatment, bonding and resin composite buildup. The set-up was incubated and kept away from light for 4 h to ensure water to continue permeating the resin-dentin interface.

After water pressure perfusion, the specimens were cut into 1 mm-thick slices under a low-speed cutter and finally polished by wet silicon carbide to grind into approximately 50 μm-thick sections containing the water perfused bonded interface. CLSM (LSM780; Carl Zeiss) was used to visualize adhesive permeation. Dyed adhesive permeation was quantified using ImageJ and expressed as a percentage of rose red fluorescence within the dentinal tubules.

#### Micro-tensile bond strength

Forty teeth from each of the control and PAMAM-OH group were cut into segments and prepared for tensile bond strength. The dentin segments were acid-etched with 37% phosphoric acid gel, washed with deionized water and gently air-dried. Specimens were then randomly divided into 2 groups (*n* = 20). The control was pre-treated with deionized water for 60 s. The experimental sample was pre-treated with 8 mg/mL PAMAM-OH for 60 s. A 2 mm-thick layer of resin composite build-up (Z250, 3 M ESPE, St. Paul, MN, USA) was placed on the dentin bonded with adhesive and light-cured. Then, specimens from each group were further divided into 2 subgroups (*n* = 10). The samples were stored in deionized water for 24 h, labelled as T0. The other samples were thermo-mechanically challenged, corresponding to 1 y of intraoral use, labelled as T1.

After storage, each bonded specimen was cut into 1 × 1 × 7 mm beams. The micro-tensile bonding test was performed by a universal testing machine with a crosshead speed of 1 mm/min. After the beam was stressed to failure, the fracture pattern of the resin-dentin interface was observed using a stereomicroscope with high magnification. Failure modes were classified as the following: adhesive failure (A), mixed failure (M), cohesive failure in resin composite (CC), and cohesive failure in dentin (CD).

### Statistical analysis

For all analyses, the significance level was set at α = 0.05. Data were exhibited as means and standard deviations (SDs). For each parameter, data sets were evaluated for their normality (Shapiro-Wilk test) and homoscedasticity assumptions (modified Levene test) prior to the use of parametric statistical methods. If the assumptions were not violated, the data sets were analyzed with one-way ANOVA or one-factor repeated-measures ANOVA, which depends on the parameter tested. Post-hoc comparisons were conducted using the Holm-Sidak procedures to identify statistical significance among groups. If those assumptions were violated, the data sets were nonlinearly transformed to satisfy those assumptions prior to performing the aforementioned statistical procedures.

## Results

### Cytotoxicity

**Fig. 1:**
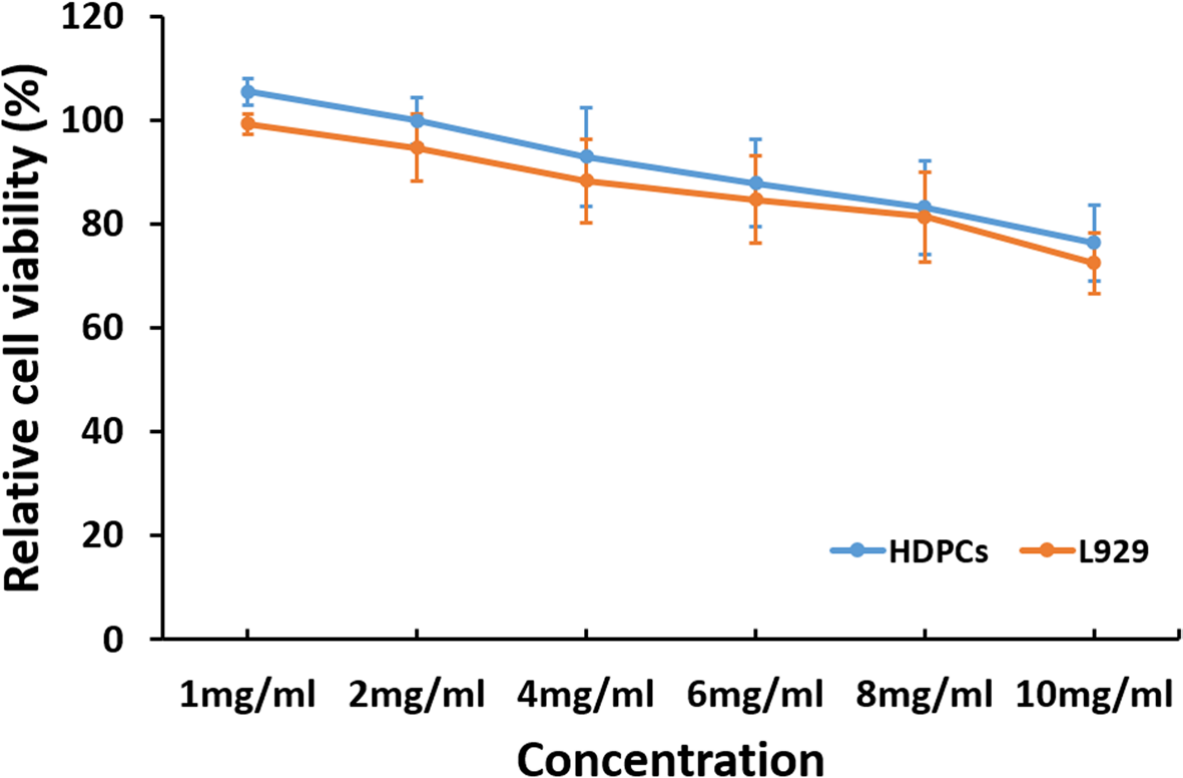
Cytotoxicity assay of PAMAM-OH to HDPCs and L929 at different concentrations by CCK-8 assay. Values are means and standard deviations

The data of the cytotoxicity assay of PAMAM-OH at various concentrations is shown in Fig. 1. For both HDPCs and L929 cells, good cell viability was manifested at concentrations from 1 to 8 mg/ml. The results demonstrated PAMAM-OH has good biocompatibility to both types of cells at working concentration.

### Characterization of adsorption capacity of PAMAM-OH on demineralized dentin

#### Adsorption capacity of PAMAM-OH on HA powders

**Fig. 2:**
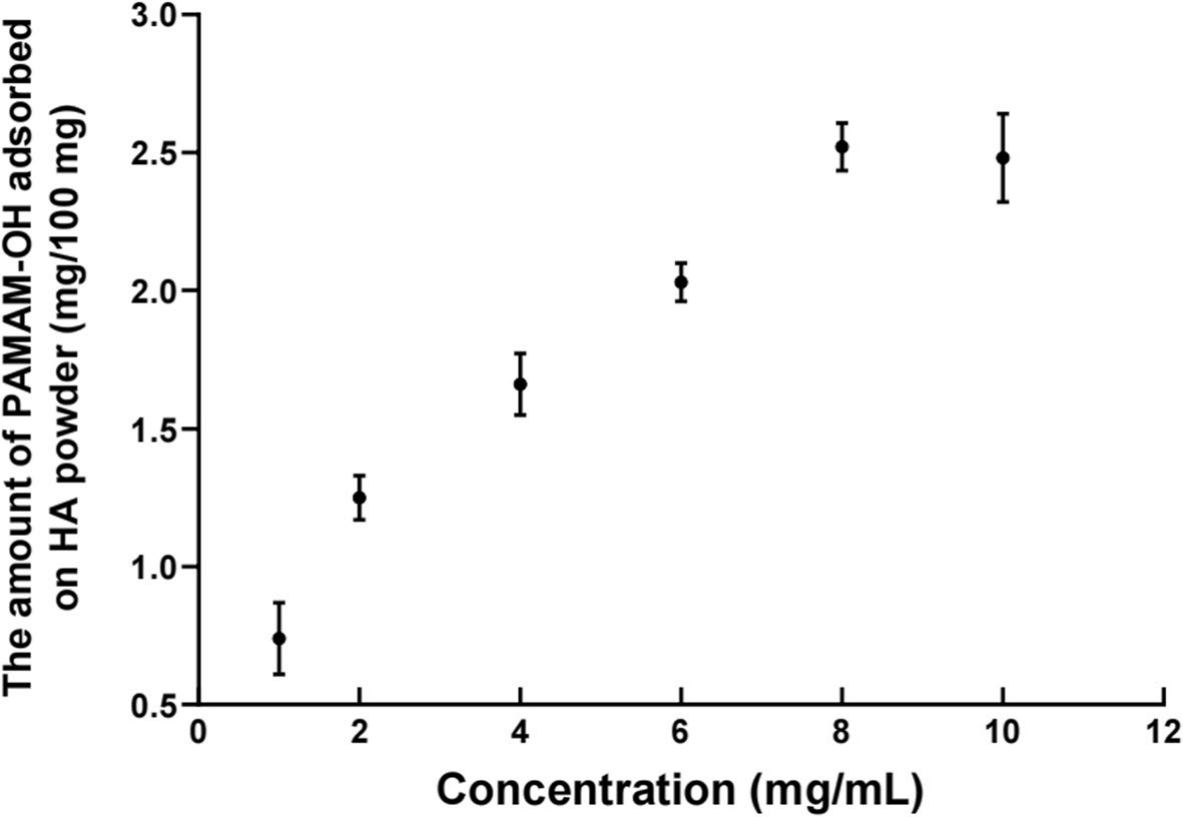
Adsorption isotherm of PAMAM-OH on HA powder (100 mg)

The quantity of adsorption tests was firstly performed to measure adsorption capacity of PAMAM-OH on HA powders. HA powders were respectively added into the PAMAM-OH solutions with different concentrations and mixed overnight. After centrifugation, the supernatant was taken out, filtered and determined at a wavelength of 282 nm. The amount of PAMAM-OH adsorbed on HA powders was calculated by the decrease of PAMAM-OH in the supernatant. The result is shown in Fig. 2**.** With the concentration of PAMAM-OH increased from 1 to 10 mg/ml, the amount of PAMAM-OH adsorbed on HA powders quickly increased at first and gradually reached saturation at 2.52 mg.

#### Assessment adsorption capacity of PAMAM-OH on demineralized dentin

**Fig. 3:**
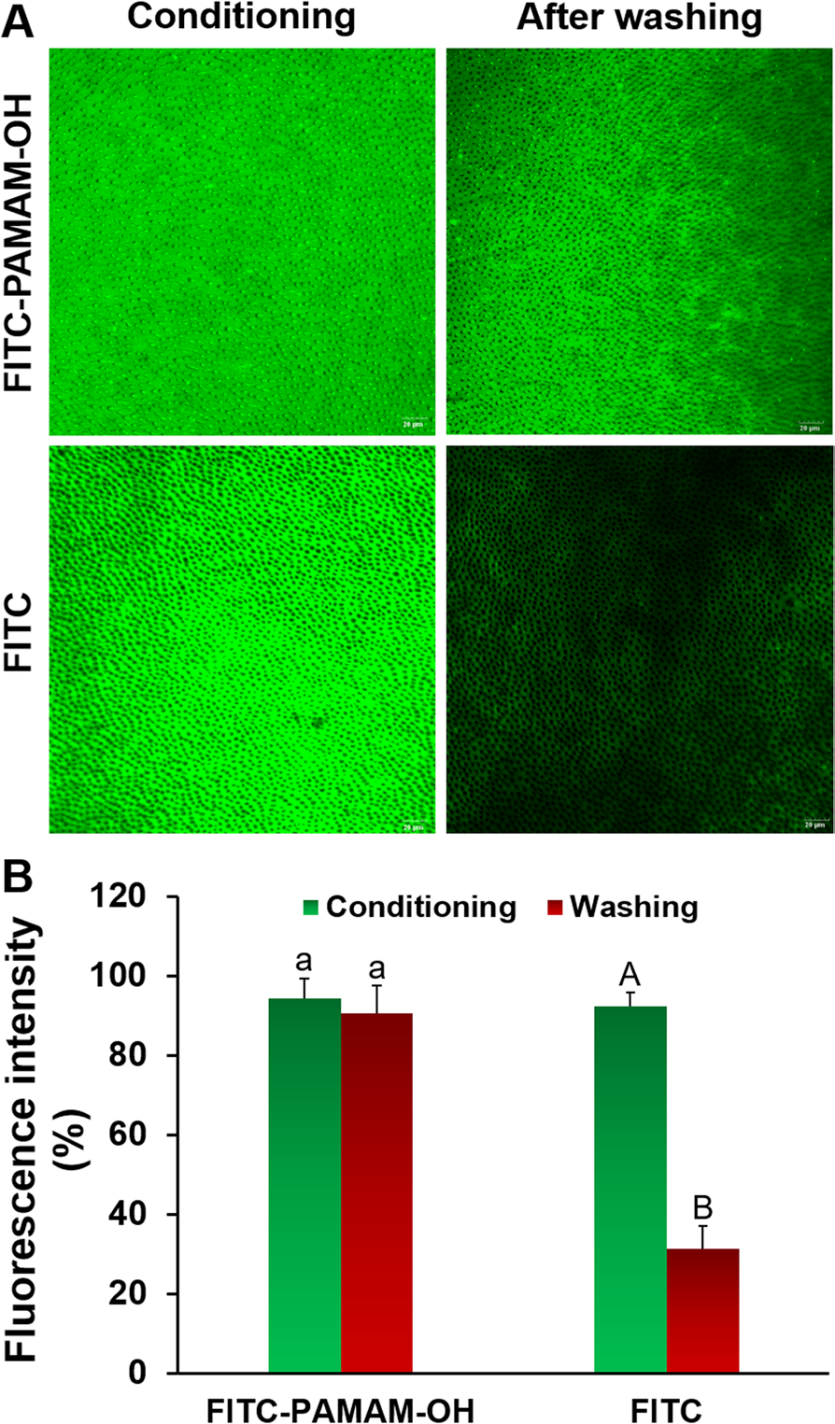
The binding capacity of PAMAM-OH to demineralized dentin. **A** The CLSM images of the demineralized dentin pretreated with FITC-labeled PAMAM-OH or free FITC, after rinsing with deionized water. **B** Relative fluorescence intensity of the two groups. Values are means and standard deviations. For FITC-PAMAM-OH group, the columns labelled with the same lowercase letters on top of the bars are not significantly different (*p* > 0.05). For FITC group, the columns labeled with the different uppercase letters are significantly different (*p* < 0.05)

Meanwhile, to investigate the ability of PAMAM-OH to adsorb on the demineralized dentin disk, the treated dentin was washed with deionized water and observed by CLSM. Figure 3A displayed that the green fluorescence was visible all over the surface of the PAMAM-OH-treated dentin. Before deionized water rinsing, both FITC-labeled PAMAM-OH group and FITC group were obviously observed intense green fluorescence, reaching 94.3% ± 5.1 and 92.2% ± 7.3%, respectively (Fig. 3B). After deionized water washing, the burning green fluorescence was similarly detected on FITC-labeled PAMAM-OH group, reaching 90.4% ± 4.4% fluorescence intensity (Fig. 3A, B). No significant difference was found in the FITC-PAMAM-OH group before and after deionized water washing (*p* > 0.05). In contrast, almost no fluorescence was observed in the FITC group and the fluorescence intensity was 31.2% ± 6.0%. These results demonstrate that PAMAM-OH has a good adsorption capacity on demineralized dentin.

### Inhibition of gelatinolytic activity

#### Inhibition of soluble rhMMP-9

**Fig. 4:**
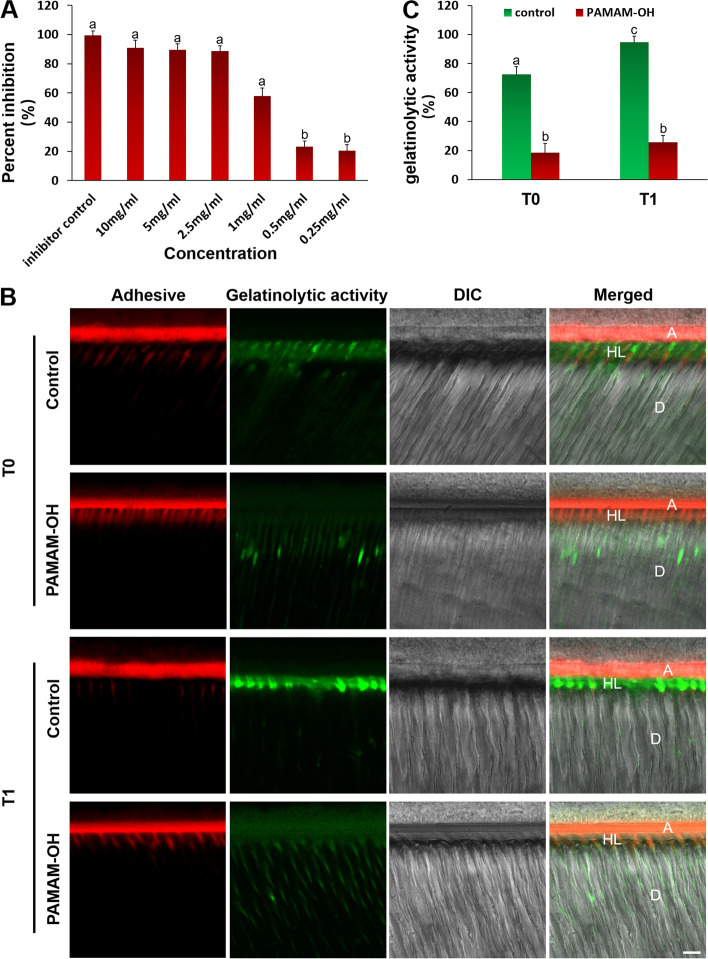
The inhibitory effect of PAMAM-OH on protease. **A** Percentage inhibition of soluble rhMMP-9 by different concentrations of PAMAM-OH. Values are means and standard deviations. Columns identified with the different lowercase letters on the top of the bars are significantly different (*p* < 0.05). **B** Representative CLSM images of in-situ zymography performed in resin-dentin interfaces pretreated with deionized water (control) or PAMAM-OH after 24 h of storage (T0) and thermal cycling (T1). Bars = 10 μm. A, adhesive layer; HL, hybrid layer; D, dentin. Red fluorescence represents adhesive and green fluorescence represents the activity of endogenous dentin gelatinase. DIC, differential interference contrast image of the resin-dentin interface. **C** The percentage gelatinolytic activity within hybrid layers after 24 h of storage (T0) and thermal cycling (T1). Values are means and standard deviations. For comparison of the four subgroups, columns identified with the different lowercase letters are significantly different (*p* < 0.05)

The influences of various concentrations of PAMAM-OH on soluble rhMMP-9 are represented in Fig. 4A. The percent inhibition of MMP-9 by PAMAM-OH with concentrations lower than 1 mg/ml was significantly lower than that of the kit inhibitor (99.4% ± 3.1%) (*p* < 0.05). There was no significant difference in the anti-MMP activities between the PAMAM-OH with concentrations equal to or higher than 1 mg/ml and the kit inhibitor (*p* > 0.05).

#### In-situ zymography of resin-dentin interface

Figure 4B exhibits the gelatinolytic zymograms formed by pretreating the dentin samples with deionized water (control group) and PAMAM-OH. Green fluorescence of the CLSM images represents the gelatinolytic activity directly within the HLs after incubation for 48 h (T0) and thermal cycling (T1), respectively. Figure 4C reports the percentage areas and fluorescence intensity of HLs in the control and PAMAM-OH groups that show green fluorescence released from the breakdown of fluorescein-conjugated gelatin. Dentin slices from control group showed intense green fluorescence along the interface, with intensity values of 72.5 ± 5.2% at T0 and 94.7 ± 6.3% at T1, respectively. By contrast, weak green fluorescence was observed in the PAMAM-OH-pretreated dentin slices, reaching 18.6 ± 4.1% at T0 and 25.8 ± 4.7% at T1, respectively. There was a significant difference in the fluorescence intensity between the control group and PAMAM-OH group regardless of incubation for 48 h (T0) or thermal cycling (T1) (*p* < 0.05) (Fig. 4B, C). After thermal cycling, the control group pretreated with deionized water showed significantly higher fluorescence intensity compared to T0 (*p* < 0.05). However, for the PAMAM-OH group, there was no significant difference in fluorescence intensity between the T1 and T0 (*p* > 0.05) (Fig. 4B, C).

#### Measurement of released ICTP

**Fig. 5:**
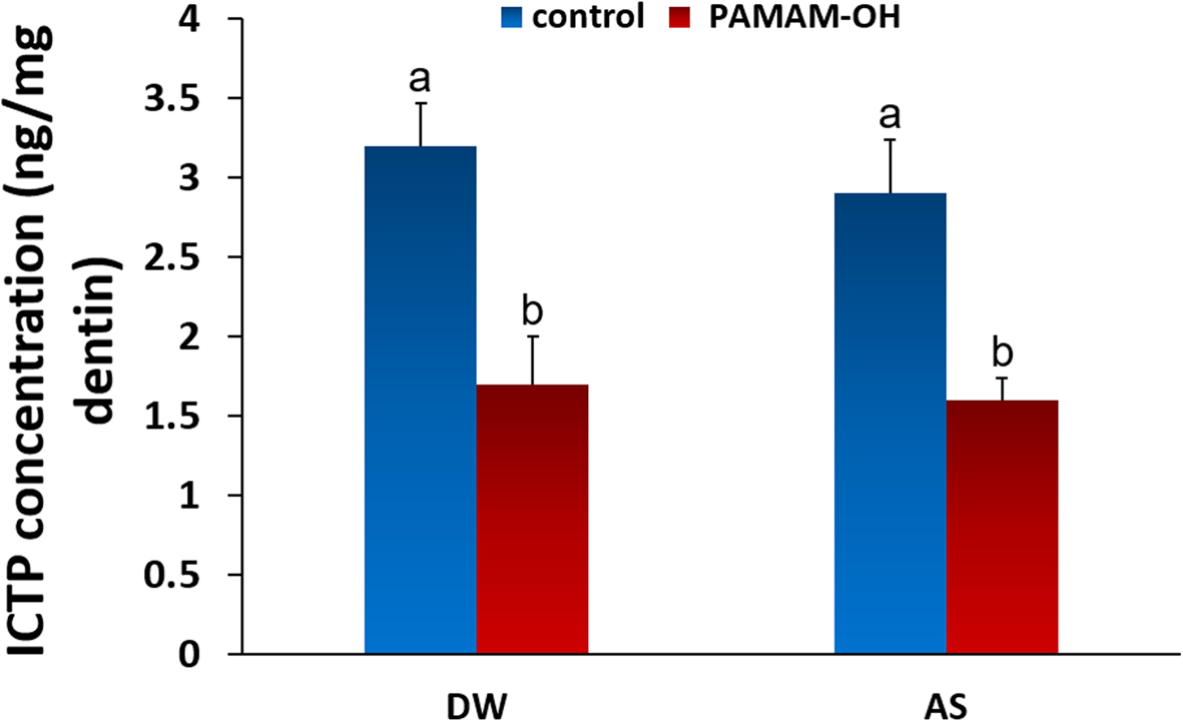
The concentration of ICTP released from demineralized dentin in control group and PAMAM-OH group after 2 weeks of incubation in deionized water (DW) and artificial saliva (AS). Data are expressed as mean ± standard deviation. For the comparison of the four subgroups, columns identified with the different lowercase letters are significantly different (*p* < 0.05)

Figure 5 summarizes the data distribution of ICTP-terminal peptides released from the demineralized dentin powder after incubation for 2 w in deionized water and artificial saliva. The ICTP concentrations of control group were 3.2 ± 0.27 ng/mL in deionized water and 2.9 ± 0.30 ng/mL in artificial saliva respectively. However, the ICTP concentrations of PAMAM-OH group were 1.7 ± 0.34 ng/mL in deionized water and 1.6 ± 0.14 ng/mL in artificial saliva, respectively. By comparison, there was a significant difference between the PAMAM-OH and control groups (*p* < 0.05). The release of the ICTP terminal telopeptides from the demineralized dentin powder was significantly correlated with the pretreatment (*p* < 0.05), rather than the type of culture medium (*p* > 0.05).

### Analysis of the effect of PAMAM-OH on resin-dentin interface

#### Adhesive permeation through resin-dentin interface

**Fig. 6:**
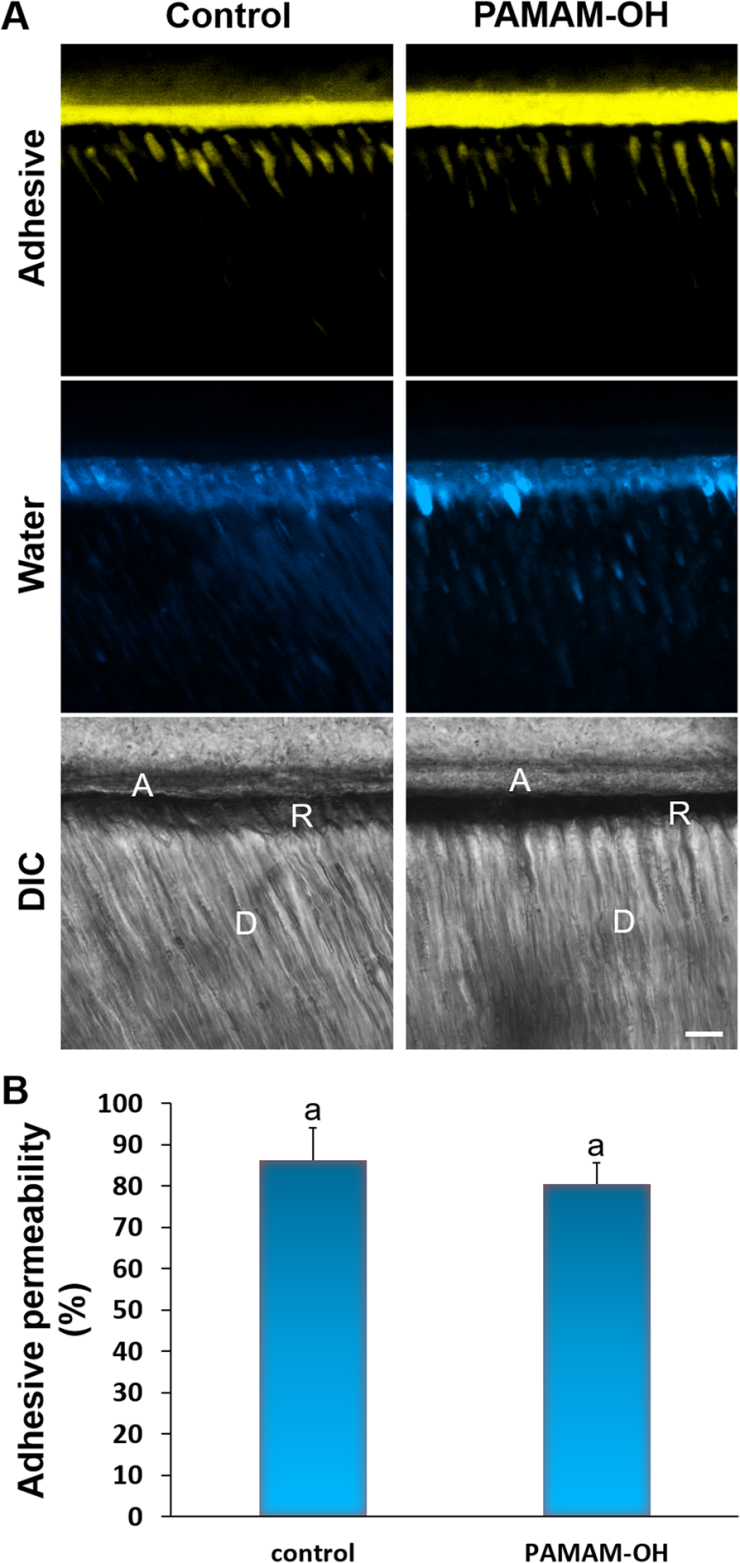
**A** Representative CLSM images showing adhesive permeability of bonded interface under simulated pulpal pressure (20 cm water pressure) in the control and PAMAM-OH groups. Bars = 10 μm. The adhesive was dyed yellow and water was dyed blue. A, adhesive layer; D, dentin; R, resin tag. DIC, differential interference contrast image of the resin-dentin interface. **B** Mean and standard deviation of the relative adhesive permeability of the resin-dentin interfaces in the deionized water control and PAMAM-OH. Columns labeled with same lowercase letters are not significantly different (*p* > 0.05)

Figure 6A shows the representative CLSM fluorescence images depicting the permeability features of the adhesive along bonded interface. The yellow channel and the blue channel represented the adhesive and water respectively. A bar chart summarizing the relative percentage of yellow fluorescence intensity is shown in Fig. 6B. Dentin slices from control group showed sufficient infiltration of yellow fluorescence along bonded interface. The relative percentages of adhesive permeability were 86.3% ± 7.8%. Likewise, the similar shape and depth of the resin tags were detected in the PAMAM-OH groups, with adhesive permeability of 80.5% ± 5.1%. The relative permeability of the PAMAM-OH pretreatment was no significant difference from that of control group (*p* > 0.05).

#### Micro-tensile bond strength test

**Fig. 7:**
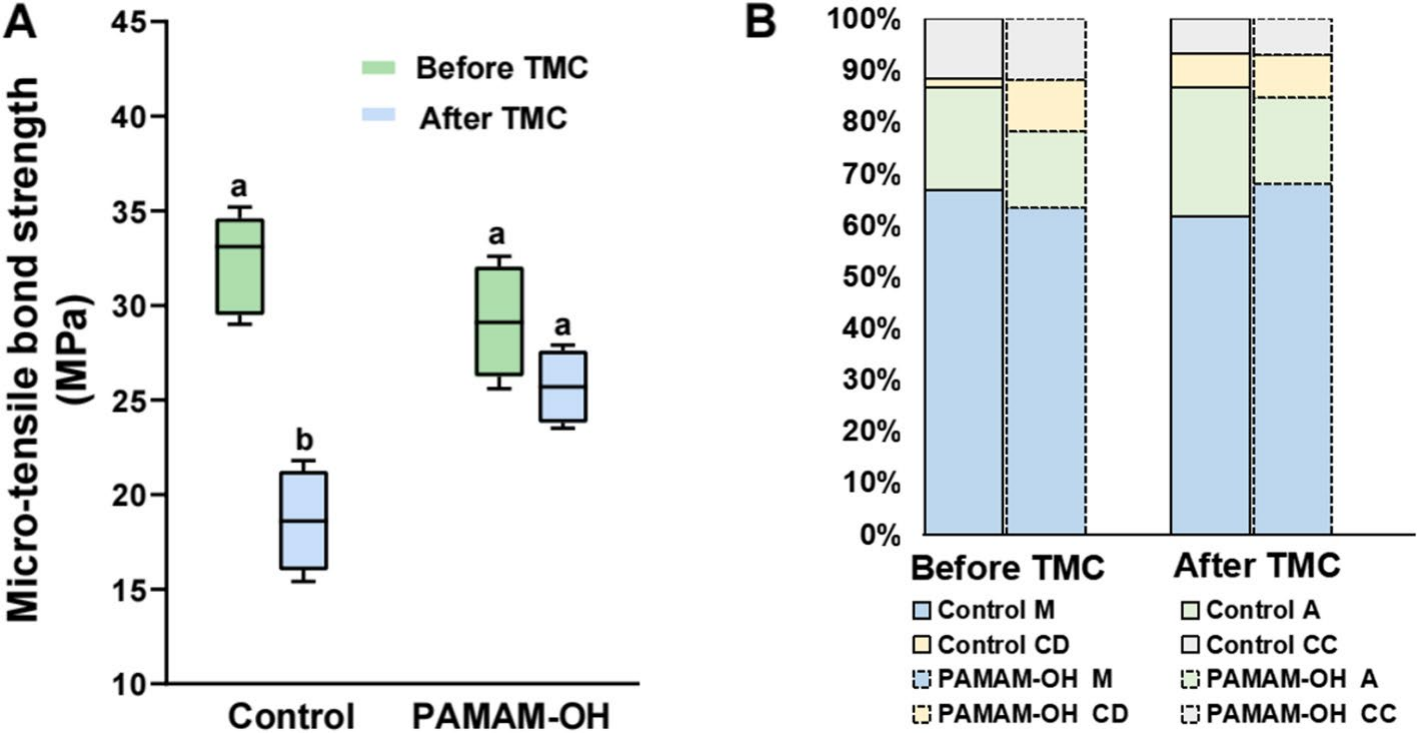
**A** Mean and standard deviation of the microtensile bond strength of dentin in the control and PAMAM-OH groups before and after thermomechanical cycling (TMC). Columns labeled with different lowercase letters are significantly different (*p* < 0.05). **B** Means of percentages of failure mode distribution in the control and PAMAM-OH groups

A bar chart summarizing the micro-tensile bond strength for each group is shown in Fig. 7A. Before thermomechanical cycling, the bond strength of the PAMAM-OH pretreatment was no significant difference from that of control group (*p* > 0.05), indicating that pretreatment with PAMAM-OH before bonding did not adversely affect the bond strength. After thermomechanical cycling, significant difference in micro-tensile bond strength of control groups was found before and after thermal cycling (*p* < 0.05). In contrast, the bond strength of PAMAM-OH pretreatment was not significantly decreased after being suffered from thermal cycling (*p* > 0.05).

The failure mode distribution of two groups is displayed in Fig. 7B. Broadly speaking, there is a correlation between the low values of bond strength and a high tendency in failure within the adhesive (A). The failure mode of all tested samples consisted mainly of mixed failures (M), with a small distribution of cohesive failure (CC or CD).

## Discussion

PAMAM-OH has been widely used to induce biomimetic remineralization on the demineralized tooth [30], but there has been no report whether it can induce intrafibrillar mineralization of dentin. Based on the size exclusion effect of fibrillar collagen where molecules with molecular weight (MW) between 6 and 40 kDa can partially access the intrafibrillar water compartments [35, 36], G4-PAMAM-OH (MW = 14.279 kDa) would access the intrafibrillar spaces and enable itself to be a desirable candidate to induce intrafibrillar remineralization to protect exposed collagen fibrils within the HLs to achieve durable resin-dentin bonds. However, the remineralization process in vivo is time-consuming, during which the denuded collagen fibrils as scaffold for the growth of mineral crystallite are inevitably vulnerable to enzymatic degradation, leading to the failure of remineralization [32]. Thereby, if PAMAM-OH (NCPs analogues) itself possesses concomitant anti-proteolytic activity during the induction of remineralization, it would be very beneficial to achieve satisfactory remineralization. In the present study, the influence of PAMAM-OH on dentin proteases was first explored, which would lay the foundation for the satisfactory intrafibrillar remineralization induced by PAMAM-OH within HLs to achieve durable resin-dentin bonds in the next work.

It is well known that the dentinal tubules are full of fluid. Intrapulpal pressure causes the intrinsic water in the pulp cavity to be continuously replenished to the dentin surface. Therefore, the adsorption capacity of PAMAM-OH on demineralized dentin is crucial for achieving its function during the remineralization process. In the study, the quantity of adsorption tests was firstly performed to measure adsorption capacity of PAMAM-OH on HA powders **(**Fig. 2**)**. With the concentration of PAMAM-OH increased from 1 to 10 mg/ml, the amount of PAMAM-OH adsorbed on HA powders quickly increased firstly and gradually reached saturation. CLSM was also performed and images **(**Fig. 3A**)** displayed that the strong green fluorescence was observed on both FITC-PAMAM-OH sample and FITC sample before washing. After washing, the strong green fluorescence was similarly detected on FITC-PAMAM-OH group with an intensity value of 90.4% ± 4.4%, while little fluorescence was detected on the control sample, reaching 31.2% ± 6.0% fluorescence intensity (Fig. 3A, B). The experimental samples withstood the deionized water rinse that simulated the pressure of intrinsic water from pulp cavity and exhibited strong fluorescence, indicating a strong adsorption capacity of PAMAM-OH on demineralized dentin. These results were also confirmed by a previous study [30]. We speculated that many anionic amide groups inside PAMAM-OH may attach to the collagen fibrils by electrostatic interaction to contribute to its adsorption capacity [30].

Dentin collagen matrix is the fundamental scaffold for the precipitation and growth of apatite mineral crystallite, which plays an important role in biomimetic remineralization [2]. It is essential to protect the exposed collagen fibrils from degrading of MMPs during remineralization. Thereby, the purpose of this paper was to investigate whether PAMAM-OH could have inhibitory effect on endogenous MMPs. The influences of various concentrations of PAMAM-OH on soluble rhMMP-9 are represented in Fig. 4A. Quantitative analysis performed by Sensolyte assay kit showed that the degree of rhMMP-9 inhibition was proportional to the concentration of PAMAM-OH. When the concentration of PAMAM-OH was equal to or higher than 1 mg/ml, the anti-MMP-9 effect was comparable to that of the kit inhibitor control group. This experiment confirmed that the activity of exogenous rhMMP-9 is inhibited by PAMAM-OH while its effect on endogenous MMP-9 embedded within dentin collagen matrix should also be explored. In this research, gelatinolytic activities of the endogenous MMP-9 directly within HLs after incubation for 48 h (T0) and thermal cycling (T1) were detected by in situ zymography. There was a significant difference in the fluorescence intensity between the control group and PAMAM-OH group regardless of incubation for 48 h (T0) or thermal cycling (T1) (*p* < 0.05) (Fig. 4B, C), indicating that PAMAM-OH has the inhibitory effect on endogenous MMPs. Additionally, the amount of type I collagen ICTP fragments was measured to determine the degradation of the demineralized collagen matrix by MMPs. The rationale for this assay was on the basis of the report that solubilized ICTP fragments are solely derived from the degradation of collagen fibrils by MMP [37]. The quantitative assay revealed that the release of ICTP fragments from the PAMAM-OH group was significantly different from that of the control group (*p* < 0.05) (Fig. 5), suggesting that PAMAM-OH has the inhibitory effect on MMPs-driven collagenolysis. Therefore, the first null hypotheses that “PAMAM-OH has no inhibitory effects on endogenous MMPs” should be rejected.

The functional mechanism of inhibitory effects of PAMAM-OH on dentin proteases remains unclear, but several factors have been speculated. The high density of nitrogen ligands in polyhydroxy-terminated dendritic polymer and the abundance of functional groups on its surface allow it to be used as a multifunctional chelating agent [38]. MMPs are classified as Ca- and Zn- dependent endoproteases [39]. PAMAM-OH may chelate Ca^2+^ and Zn^2+^ that bind to the Zn^2+^- and Ca^2+^- active sites of the catalytic domain of MMPs, spatially intercepting the active sites and restraining the activity of MMPs. Besides, the previous study has reported that PAMAM-COOH may electrostatically bind to proteins and inhibit the activity of MMPs [40]. Therefore, we surmise that negatively-charged PAMAM-OH may also electrostatically bind to positively-charged domain of MMPs, which contribute to its inhibitory effect.

Sufficient infiltration of resin monomers is important for the stability of the bonded interface. The dentin samples pretreated with PAMAM-OH were observed by CLSM to evaluate whether it has adverse effect on adhesive infiltration [41]. The CLSM images and quantitative analysis indicated that PAMAM-OH pretreatment did not decline the permeability of adhesive (Fig. 6A, B).

The microtensile bond strength was also conducted to assess if pretreatment of PAMAM-OH has adverse effect on the dentin bond strength. The result of bond strength indicated that pretreatment of PAMAM-OH on dentin did not decline the immediate tensile bond strengths. However, the bond strength of PAMAM-OH group was significantly higher than that of the control after aging (*p* < 0.05) (Fig. 7A), indicating pretreatment of PAMAM-OH prolonged the resin-dentin bonds. These findings were confirmed by the results got from in situ zymography after aging. PAMAM-OH possesses anti-proteolytic activity and prevents exposed collagen fibrils within HLs from enzymatic degradation, which may conduce to the improvement of the durability of resin-dentin bonds. Hence, the second null hypotheses that “PAMAM-OH has adverse impact on the performances of resin-dentin bonds” should be rejected.

Further studies on the interaction between PAMAM-OH and dentin matrix, and studies of intrafibrillar remineralization induced by PAMAM-OH within HLs are required to support its potential clinical application.

## Conclusions

With the limitations of present study, it may be summarized that PAMAM-OH with low cytotoxicity possesses anti-proteolytic activity and prevents exposed collagen fibrils within HLs from degradation, which lays the foundation for the satisfactory intrafibrillar remineralization induced by PAMAM-OH within HLs to achieve durable resin-dentin bonds in the next work.

## Data Availability

The original contributions presented in the study are included in the article. Further inquiries can be directed to the corresponding authors.

## Abbreviations

HLs: hybrid layers
PAMAM-OH: polyhydroxy-terminated poly(amidoamine) dendrimer
MMPs: matrix metalloproteinases
NCPs: non-collagenous proteins
PAMAM: Poly(amidoamine)
HDPCs: human dental pulp stem cells
CCK-8: Cell Counting Kit-8
DMEM: Dulbecco’s Modified Eagle’s Medium
HA: hydroxyapatite
ICTP: Cross-linked carboxyterminal telopeptide
SDs: standard deviations
A: adhesive failure
M: mixed failure
CC: cohesive failure in resin composite
CD: cohesive failure in dentin
ANOVA: Analysis of variances
